# Acute and chronic toxicities of *Bacopa monnieri* extract in Sprague-Dawley rats

**DOI:** 10.1186/s12906-016-1236-4

**Published:** 2016-07-27

**Authors:** Seewaboon Sireeratawong, Kanjana Jaijoy, Parirat Khonsung, Nirush Lertprasertsuk, Kornkanok Ingkaninan

**Affiliations:** 1 Department of Pharmacology, Faculty of Medicine, Chiang Mai University, Chiang Mai, 50200 Thailand; 2 Division of Pharmacology, Department of Preclinical Science, Faculty of Medicine, Thammasat University, Pathum Thani, 12120 Thailand; 3 McCormick Faculty of Nursing, Payap University, Chiang Mai, 50000 Thailand; 4 Department of Pathology, Faculty of Medicine, Chiang Mai University, Chiang Mai, 50200 Thailand; 5 Bioscreening Unit, Department of Pharmaceutical Chemistry and Pharmacognosy, Faculty of Pharmaceutical Sciences and Centre of Excellence for Innovation in Chemistry, Naresuan University, Phitsanulok, 65000 Thailand

**Keywords:** *Bacopa monnieri*, Acute toxicity, Chronic toxicity

## Abstract

**Background:**

*Bacopa monnieri* is a medicinal plant which has long been used in Ayurvedic medicines to augment brain function and to improve memory. The purpose of our study was to identify and evaluate possible toxic effects of *B. monnieri* extract in rats by assessing hematological, biochemical, and histopathological parameters.

**Methods:**

Acute oral toxicity of *Bacopa monnieri* extract was studied in female rats by giving a single orally administered dose at a level of 5,000 mg/kg. The rats were monitored for toxic signs for 14 days. In the chronic toxicity test, groups of both female and male rats were given daily oral doses of *B. monnieri* extract at dose levels of either 30, 60, 300 or 1,500 mg/kg for 270 days. The behavior and health of the animals was then monitored. At the end of the observation period, the body and organ weights of the rats in each group were measured. Blood was collected and necropsy was performed to evaluate their hematology, blood clinical chemistry, and microanatomy.

**Results:**

The acute toxicity test found no significant differences between the experimental and the control group rats. In the chronic toxicity test, animal behavior and health of the experimental groups were normal, just as in the control rats. All values of other parameters assessed remained within the normal range.

**Conclusion:**

A single oral administration of *B. monnieri* extract at the dose of 5,000 mg/kg did not cause any serious undesirable effects. *B. monnieri* extract at doses of 30, 60, 300 and 1,500 mg/kg given for 270 days did not produce any toxicity in rats.

## Background

*Bacopa monnieri* (L.) Wettst, known as Brahmi or water hyssop, (Family Plantaginaceae), is a perennial creeper found in marshy areas throughout Asia where it is used in Ayurvedic medicine for enhancing memory and improving brain function [1]. It has recently been reported that *B. monnieri* extract has several pharmacological activities, e.g., a neuroprotective effect [2, 3], ameliorating cognitive dysfunction [4–6], increasing cerebral blood flow [7], enhancing the activity of antioxidant enzymes and intracellular signaling pathways [8], an antiparkinsonian agent [9], reducing blood pressure [10], hepatoprotection [11], anti-fertility [12], anti-addiction [13], antioxidant [14, 15], antidepressant [16], anti-stress [17], anti-ulcer [18], anti-cancer [19], and anti-inflammation [20].

Clinical studies have shown that *B. monnieri* reduces the rate of memory loss of newly acquired information [21], improves memory performance in older persons [22], and enhances cognitive performance in humans [23]. However, *B. monnieri* was also shown to cause side effects in the gastrointestinal tract, i.e., nausea, increased stool frequency and abdominal cramps [22]. Severe liver toxicity has been reported in a woman after taking several Ayurvedic herbs, including *B. monnieri,* for nine months; however, after she stopped taking the herbs, her liver function returned to normal [24]. Because of the potential positive ethnobotanical and pharmacological applications of the plant, this study was designed to investigate possible toxic effects of the *B. monnieri* extract in rats.

## Methods

### Plant material and extract

The *B. monnieri* was collected in Phetchaburi Province, Thailand, and was identified by Prof. Dr. Wongsatit Chuakul. The voucher specimen (Phrompittayarat 001) is maintained at the PBM Herbarium, Mahidol University, Thailand. An aerial portion of *B. monnieri* was cut into small pieces, dried (50 °C, 12 h.) and then pulverized. The dried powder was percolated twice with 95 % ethanol at the ratio of 1 g: 7 ml. The percolate was dried in a rotary evaporator under reduced pressure. The yield was 10 % of the fresh weight. The phytochemical study found the extract contained 6.25 % (w/w) of total saponins, a mixture of 0.87 % of bacoside A_3_, 1.03 % of bacopaside I, 1.82 % of bacopaside II, 0.8 % of bacopaside X, and 1.73 % of bacopasaponin C. [25, 26]. The extract was kept in a dark bottle in the refrigerator at 5 °C until used.

### Animals

Male and female Sprague-Dawley rats, 4–5 week-old and weighing 200–250 g, were purchased from the National Laboratory Animal Center (NLAC), Nakorn Pathom, Thailand. All rats were kept in an animal room at a controlled temperature of 25 ± 1 °C and with a 12 h light-dark cycle. A standard rat chow and water ad libitum were provided. The animals were acclimatized for one week before the start of the experiment. The experimental protocols were approved by the Animal Ethics Committee of Faculty of Medicine, Thammasat University (AE011/2552).

### Acute toxicity test

Following OECD Test Guideline 420, in the sighting study female Sprague-Dawley rats received a starting dose of 2,000 mg/kg of extract (Annex 2 - Flow chart of the sighting study), but no toxicity was observed. A main study starting dose of 5,000 mg/kg was the limit in the acute toxicity study. The female rats were randomly divided into control and treatment groups of five rats each. Rats were fasted overnight prior to dosing on day 1. Group 1, the control group, received distilled water (1 mL/kg) and group 2, the test group, received *B. monnieri* extract at a dose of 5,000 mg/kg in a constant volume of 1 mL/kg body weight by oral administration.

Observation of toxic signs was recorded systematically for the first 30 min, then periodically during the first 24 h. At the end of the experiment the surviving rats were kept for further 14 days to allow daily observation of clinical signs of toxicity. The body weight of the animals was measured prior to dosing and then weekly after that. On day 15, the surviving animals were sacrificed using an intraperitoneal overdose of pentobarbital sodium. Their internal organs, including heart, lungs, liver, kidney, spleen, adrenal glands, sex organs and brain, were excised, and weighed and a gross pathological examination was conducted. The organs were then fixed in a 10 % neutral buffered formaldehyde solution for histopathological examination [27, 28].

### Chronic toxicity test

The chronic toxicity test was conducted in accordance with WHO and OECD guidelines. The usual dose of *B. monnieri* in food supplements for humans is 300 mg/day or 5 mg/kg/day (the average body weight of a Thai adult is about 60 kg). The dosage of the plant extract used in rats, approximately 30 mg/kg/day, was calculated based on body surface area [30]. Doses of 30, 60, 300 and 1,500 mg/kg/day of *B. monnieri* extract were used for the chronic toxicity evaluation. The rats were divided by sex into six groups, ten male and ten female each. All the rats were orally administered either distilled water (control group) or *B. monnieri* extract once daily for 270 days (experimental group). A satellite group received a dose of *B. monnieri* extract of 1,500 mg/kg per day for 270 days after which the rats were then reared for an additional 28 days with no *B. monnieri* extract to observe their recovery and to identify any delayed effects of the extract. Animals were weighed and observed daily for toxicological signs, physiological and behavioral changes, as well as mortality. The animals were anesthetized with an intraperitoneal injection of pentobarbital sodium at the end of the experiment. Blood samples were collected from the common carotid artery with an EDTA tube for hematological study and with a non-EDTA tube for biochemical study. Hematological analysis using standard techniques was performed for red blood cell count (RBC), hemoglobin (HB), hematocrit (HCT), mean corpuscular volume (MCV), mean corpuscular hemoglobin (MCH), mean corpuscular hemoglobin concentration (MCHC), platelet count (PLT), white blood cell count (WBC) as well as neutrophil (PMN), lymphocyte (LYMP), monocyte (MONO), eosinophil (EOS), and basophil (BASO) concentrations. Blood samples were biochemically analyzed for levels of glucose (GLU), creatinine (CRE), blood urea nitrogen (BUN), total protein (TP), albumin (ALB), total bilirubin (T-BIL), direct bilirubin (D-BIL), alanine aminotransferase (ALT), aspartate aminotransferase (AST), and alkaline phosphatase (ALP). Finally, the rats were sacrificed and their internal organs were excised, observed, weighed and fixed in 10 % neutral buffered formaldehyde solution for further pathological examination [28, 29].

### Statistical analysis

One-way analysis of variance (ANOVA) and Dunnett’s test were performed to determine statistical significance using the SPSS program (version 22.0). In the acute toxicity study, the data were analyzed using Student’s *t*-test. Results are expressed as mean ± standard error of mean (S.E.M.). *P* values less than 0.05 were considered significant.

## Results

**Fig. 1:**
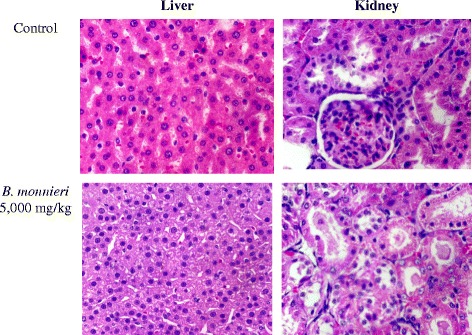
Histopathology of the liver and kidney of rats in acute toxicity test at 40x magnification (haematoxylin-eosin stain)

A large single oral dose of *B. monnieri* extract (5,000 mg/kg) did not cause mortality in rats. Normal behavioral patterns were observed and no changes of eyes, skin, fur, mucous membranes, respiration, circulatory system, autonomic or central nervous systems were found after that dose. The body weight on day 14 of the extract-treated rats had significantly increased compared to that of the control group (Table 1). As shown in Table 2, the average lung weight of treated rats was significantly greater than in the control group, although physical appearance (color and texture) was normal. No significant histopathological change in the internal organs, including the liver and the kidney, were observed (Fig. 1).

**Table 1:** Body weight of female rats in acute toxicity test

	Body weight (g)		
	Day 0	Day 7	Day 14
Control	218.00 ± 3.74	215.00 ± 2.24	233.00 ± 2.00
*B. monnieri* extract 5,000 mg/kg	234.00 ± 4.30	255.00 ± 6.12	260.00 ± 7.58*

Values are expressed as mean ± S.E.M., *n* = 10

*Significantly different from control, *p* < 0.05

**Table 2:** Organ weight (g) of female rats in acute toxicity test

	Organ weight (g)	
	Control	*B. monnieri* extract 5,000 mg/kg
Brain	1.79 ± 0.08	1.73 ± 0.03
Lung	1.27 ± 0.02	1.44 ± 0.06*
Heart	0.84 ± 0.05	0.92 ± 0.03
Liver	6.74 ± 0.29	9.10 ± 0.61
Pancreas	0.62 ± 0.04	0.86 ± 0.08
Spleen	0.65 ± 0.04	0.82 ± 0.04
Adrenal	0.04 ± 0.00	0.04 ± 0.00
kidney	0.83 ± 0.02	0.86 ± 0.02
Ovary	0.07 ± 0.00	0.07 ± 0.00
Uterus	0.49 ± 0.02	0.47 ± 0.04

Values are expressed as mean ± S.E.M., *n* = 10

*Significantly different from control, *p* < 0.05

**Fig. 2:**
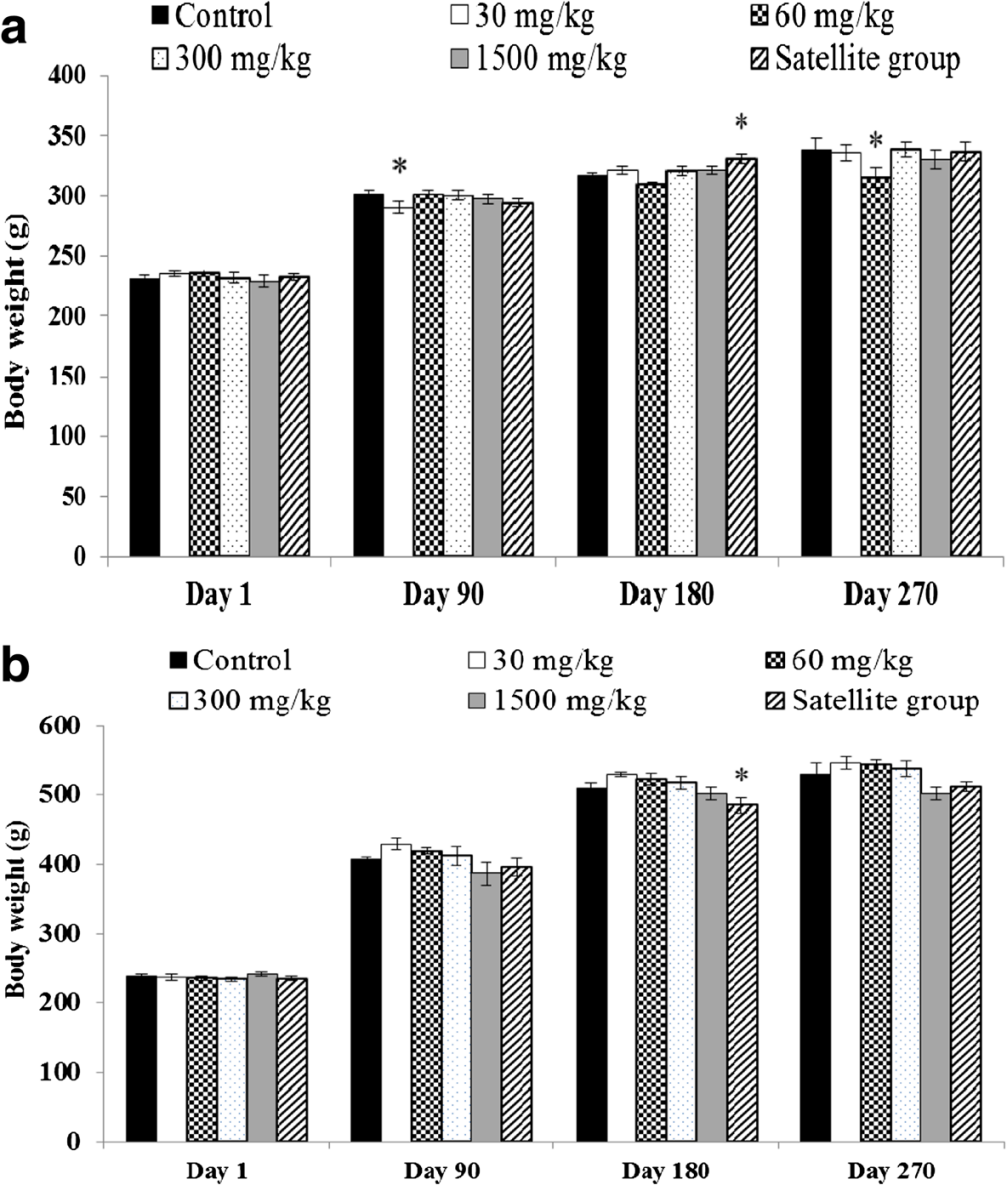
Body weight of rats treated with *B. monnieri* in chronic toxicity. **a** Female. **b** Male. *Significantly different from control, *p* < 0.05

In the chronic toxicity study (Fig. 2) a small but significant decrease in the body weight of female rats was observed in the experimental group with some doses of extract compared to the control group (distilled water) measured on the same day. The organ weight of female rats is presented in Table 3. Female *B. monnieri* extract-treated rats showed the significant greater in the weight of the brain (300 and 1,500 mg/kg), pancreas (1,500 mg/kg) and ovary (60, 300 and 1,500 mg/kg), while a significant smaller in liver weight (60 mg/kg) was observed. In male *B. monnieri* extract-treated rats, significant greater in the weight of the lungs (1,500 mg/kg), kidneys (30 mg/kg), and epididymes (60 and 300 mg/kg) were observed. The weight of the heart (satellite group) and liver (60 and 300 mg/kg) was significantly lower in male extract-treated groups than in the control group (Table 4).

**Table 3:** Organ weight (g) of female rats in chronic toxicity test

	Control			*B. monnieri* extract														
				30 mg/kg			60 mg/kg			300 mg/kg			1,500 mg/kg			Satellite group		
Brain	1.97	±	0.02	1.94	±	0.03	1.94	±	0.03	2.07	±	0.04*	2.08	±	0.02*	2.01	±	0.03
Lung	2.16	±	0.26	2.16	±	0.26	2.00	±	0.14	2.14	±	0.13	2.32	±	0.19	2.00	±	0.13
Heart	1.27	±	0.05	1.22	±	0.03	1.24	±	0.04	1.38	±	0.09	1.36	±	0.06	1.31	±	0.05
Liver	9.90	±	0.32	9.55	±	0.28	8.71	±	0.30*	9.72	±	0.23	9.33	±	0.29	9.54	±	0.27
Spleen	0.83	±	0.03	0.81	±	0.03	0.74	±	0.03	0.83	±	0.03	0.84	±	0.04	0.79	±	0.04
Pancreas	1.03	±	0.09	1.09	±	0.08	1.21	±	0.14	1.34	±	0.13	1.42	±	0.14*	0.98	±	0.12
Adrenal	0.04	±	0.00	0.04	±	0.00	0.05	±	0.00	0.04	±	0.00	0.04	±	0.00	0.04	±	0.00
kidney	1.27	±	0.02	1.28	±	0.02	1.20	±	0.03	1.22	±	0.02	1.24	±	0.04	1.25	±	0.03
Ovary	0.07	±	0.00	0.08	±	0.00	0.09	±	0.01*	0.09	±	0.01*	0.09	±	0.01*	0.07	±	0.00
Uterus	1.18	±	0.14	1.36	±	0.33	1.01	±	0.07	0.96	±	0.09	0.86	±	0.05	1.13	±	0.10

A satellite group was given the *B. monnieri* extract at 1,500 mg/kg daily over 270 days followed by no treatment for 28 days

Values are expressed as mean ± S.E.M., *n* = 10. *Significantly different from control, *p* < 0.05

**Table 4:** Organ weight (g) of male rats in chronic toxicity test

	Control			*B. monnieri* extract														
				30 mg/kg			60 mg/kg			300 mg/kg			1,500 mg/kg			Satellite group		
Brain	2.11	±	0.03	2.16	±	0.03	2.16	±	0.04	2.14	±	0.04	2.17	±	0.04	2.11	±	0.03
Lung	2.34	±	0.10	2.60	±	0.13	2.58	±	0.14	2.58	±	0.20	3.59	±	0.21*	2.36	±	0.13
Heart	1.84	±	0.07	1.89	±	0.05	1.76	±	0.03	1.79	±	0.06	1.97	±	0.07	1.67	±	0.06*
Liver	15.80	±	0.44	16.80	±	0.34	14.22	±	0.23*	14.09	±	0.56*	14.61	±	0.61	14.75	±	0.37
Pancreas	0.97	±	0.06	1.05	±	0.04	0.99	±	0.04	1.05	±	0.04	0.98	±	0.04	0.96	±	0.03
Spleen	1.27	±	0.04	1.21	±	0.12	1.41	±	0.14	1.08	±	0.14	1.54	±	0.13	0.94	±	0.09
Adrenal	0.04	±	0.00	0.04	±	0.00	0.05	±	0.00	0.04	±	0.00	0.04	±	0.00	0.04	±	0.00
Kidney	1.86	±	0.04	2.03	±	0.06*	1.82	±	0.04	1.76	±	0.04	1.76	±	0.06	1.74	±	0.03
Testis	2.13	±	0.03	2.11	±	0.09	2.11	±	0.03	2.13	±	0.03	2.05	±	0.07	2.00	±	0.04
Epididymis	0.86	±	0.02	0.93	±	0.03	0.99	±	0.03*	1.04	±	0.06*	0.93	±	0.03	0.87	±	0.02

A satellite group was given the *B. monnieri* extract at 1,500 mg/kg daily over 270 days followed by no treatment for 28 days

Values are expressed as mean ± S.E.M., *n* = 10. *Significantly different from control, *p* < 0.05

The effect of *B. monnieri* extract on hematological parameters is depicted in Tables 5 and 6. In the female satellite group, the values of RBC, HB, HCT, and MCV were significantly increased. However, MCH, MCHC and PLT levels were significantly lower than those of the control group. Moreover, the amounts of WBC and LYMP were significantly increased in female rats treated with *B. monnieri* extract at doses of 1,500 and 300 mg/kg, respectively. In the male extract-treated groups, the levels of RBC (30 mg/kg) and MCHC (satellite group) were significantly decreased, whereas the level of HCT was significantly increased in the satellite group. The amount of WBC, NEU and LYMP was significantly increased in male rats treated with *B. monnieri* extract at doses of 300 and 1,500 mg/kg.

**Table 5:** Hematological parameters of female rats in chronic toxicity test

Hematological parameter	Control			*B. monnieri* extract														
				30 mg/kg			60 mg/kg			300 mg/kg			1,500 mg/kg			Satellite group		
RBC (x10^6^/μl)	8.96	±	0.27	9.03	±	0.37	8.80	±	0.13	8.56	±	0.20	8.98	±	0.18	9.92	±	0.20*
HB (g/dl)	16.36	±	0.37	16.22	±	0.30	16.24	±	0.22	15.84	±	0.29	16.41	±	0.27	17.50	±	0.26*
HCT (%)	50.00	±	1.60	50.80	±	2.12	48.80	±	0.73	48.60	±	1.05	50.20	±	0.89	56.60	±	0.98*
MCV (fl)	55.70	±	0.30	56.10	±	0.43	55.40	±	0.27	56.50	±	0.31	55.80	±	0.59	57.00	±	0.30*
MCH (pg)	18.30	±	0.22	18.09	±	0.44	18.41	±	0.25	18.56	±	0.27	18.30	±	0.31	17.61	±	0.13
MCHC (g/dl)	32.83	±	0.45	32.18	±	0.79	33.13	±	0.38	32.82	±	0.37	32.75	±	0.38	30.90	±	0.17*
PLT (x10^5^/μl)	0.83	±	0.05	0.85	±	0.05	0.77	±	0.02	0.73	±	0.02	0.72	±	0.09	0.68	±	0.04*
WBC (x10^3^/μl)	5.18	±	0.34	4.96	±	0.38	5.87	±	0.36	6.32	±	0.61	7.03	±	0.63*	6.27	±	0.67
NEU (x10^3^/μl)	1.37	±	0.16	1.28	±	0.15	1.30	±	0.14	1.53	±	0.42	1.41	±	0.19	2.03	±	0.26
LYMP (x10^3^/μl)	3.18	±	0.39	3.40	±	0.24	4.27	±	0.28	9.59	±	5.25*	5.31	±	0.50	3.63	±	0.58
MONO (x10^3^/μl)	0.15	±	0.03	0.23	±	0.06	0.21	±	0.06	0.27	±	0.07	0.19	±	0.03	0.23	±	0.04
EOS (x10^3^/μl)	0.04	±	0.02	0.04	±	0.03	0.09	±	0.03	0.04	±	0.02	0.06	±	0.02	0.03	±	0.02
BASO (x10^3^/μl)	0.00	±	0.00	0.00	±	0.00	0.00	±	0.00	0.00	±	0.00	0.00	±	0.00	0.00	±	0.00

A satellite group was given the *B. monnieri* extract at 1,500 mg/kg daily over 270 days followed by no treatment for 28 days

*RBC* red blood cell count, *HB* hemoglobin, *HCT* hematocrit, *MCV* mean corpuscular volume, *MCH* mean corpuscular hemoglobin, *MCHC* mean corpuscular hemoglobin concentration, *PLT* platelet count, *WBC* white blood cell count, *NEU* neutrophil, *LYMP* lymphocyte, *MONO* monocyte, *EOS* eosinophil, *BASO* basophil

Values are expressed as mean ± S.E.M., *n* = 10. *Significantly different from control, *p* < 0.05

**Table 6:** Hematological parameters of male rats in chronic toxicity test

Hematological parameter	Control			*B. monnieri* extract														
				30 mg/kg			60 mg/kg			300 mg/kg			1,500 mg/kg			Satellite group		
RBC (x10^6^/μl)	9.76	±	0.18	8.53	±	0.89*	10.03	±	0.30	9.53	±	0.30	9.60	±	0.21	10.73	±	0.20
HB (g/dl)	16.99	±	0.23	16.73	±	0.50	16.92	±	0.43	16.73	±	0.51	16.77	±	0.41	17.82	±	0.28
HCT (%)	52.00	±	0.91	51.00	±	1.86	52.90	±	1.55	50.60	±	1.58	50.50	±	1.30	57.50	±	0.98*
MCV (fl)	53.00	±	0.21	53.40	±	0.31	52.60	±	0.27	53.00	±	0.30	52.50	±	0.27	53.50	±	0.31
MCH (pg)	17.38	±	0.17	17.54	±	0.09	16.85	±	0.15	17.54	±	0.11	16.41	±	1.03	16.56	±	0.12
MCHC (g/dl)	32.80	±	0.28	32.91	±	0.26	32.07	±	0.25	33.16	±	0.19	33.18	±	0.22	30.92	±	0.12*
PLT (x10^5^/μl)	0.95	±	0.03	0.86	±	0.03	0.97	±	0.03	0.93	±	0.04	1.85	±	0.89	0.89	±	0.04
WBC (x10^3^/μl)	7.70	±	0.42	7.23	±	0.69	9.52	±	0.40	11.62	±	1.44*	10.01	±	0.72*	7.79	±	0.60
NEU (x10^3^/μl)	1.37	±	0.16	1.80	±	0.23	2.12	±	0.22	2.89	±	1.19*	2.18	±	0.26	2.10	±	0.26
LYMP (x10^3^/μl)	5.94	±	0.28	5.14	±	0.52	6.90	±	0.29	8.08	±	0.50*	7.31	±	0.50*	5.31	±	0.55
MONO (x10^3^/μl)	0.27	±	0.06	0.21	±	0.07	0.30	±	0.07	0.49	±	0.17	0.45	±	0.12	0.35	±	0.06
EOS (x10^3^/μl)	0.11	±	0.05	0.08	±	0.03	0.20	±	0.08	0.06	±	0.05	0.07	±	0.03	0.02	±	0.02
BASO (x10^3^/μl)	0.00	±	0.00	0.00	±	0.00	0.00	±	0.00	0.00	±	0.00	0.00	±	0.00	0.00	±	0.00

A satellite group was given the *B. monnieri* extract at 1,500 mg/kg daily over 270 days followed by no treatment for 28 days

*RBC* red blood cell count, *HB* hemoglobin, *HCT* hematocrit, *MCV* mean corpuscular volume, *MCH* mean corpuscular hemoglobin, *MCHC* mean corpuscular hemoglobin concentration, *PLT* platelet count, *WBC* white blood cell count, *NEU* neutrophil, *LYMP* lymphocyte, *MONO* monocyte, *EOS* eosinophil, *BASO* basophil

Values are expressed as mean ± S.E.M., *n* = 10. *Significantly different from control, *p* < 0.05

Clinical blood chemistry parameters were used to clarify the effect of *B. monnieri* extract on the liver (SGPT, SGOT, TP, ALB, ALP, T-BIL and D-BIL), kidney (BUN and CRE), and pancreatic (GLU) function. As shown in Table 7, the level of GLU was significantly decreased in female rats treated with *B. monnieri* extract at doses of 60, 300 and 1,500 mg/kg. A small but significant increase of CRE levels and a decrease of TP levels were observed in the female satellite group. In male rats, the level of AST was significantly decreased in groups treated with *B. monnieri* extract at doses of 60, 300 and 1,500 mg/kg. In the male satellite group, a small but significant increase of BUN, ALT and ALP levels was found when compared to those of the control group.

**Table 7:** Biochemical parameters of female and male rats in chronic toxicity test

Biochemical parameter	Control			*B. monnieri* extract														
				30 mg/kg			60 mg/kg			300 mg/kg			1,500 mg/kg			Satellite group		
Female rats																		
GLU (mg/dl)	177.30	±	5.66	159.90	±	7.50	147.50	±	6.55*	134.10	±	8.50*	132.20	±	7.96*	170.3	±	8.85
BUN (mg/dl)	16.12	±	1.99	16.54	±	0.41	15.76	±	1.23	16.47	±	0.44	27.50	±	11.31	21.32	±	0.53
CRE (mg/dl)	0.56	±	0.02	0.57	±	0.01	0.55	±	0.01	0.58	±	0.01	0.60	±	0.02	0.61	±	0.02*
TP (g/dl)	6.29	±	0.08	6.20	±	0.08	6.15	±	0.09	6.22	±	0.12	6.16	±	0.10	5.86	±	0.03*
ALB (g/dl)	3.20	±	0.03	3.27	±	0.03	3.27	±	0.03	3.20	±	0.05	3.12	±	0.04	3.18	±	0.05
T-BIL (mg/dl)	0.10	±	0.01	0.10	±	0.01	0.11	±	0.01	81.18	±	81.09	0.10	±	0.00	0.25	±	0.02
D-BIL (mg/dl)	0.18	±	0.02	0.17	±	0.02	0.23	±	0.03	0.28	±	0.08	0.22	±	0.03	0.11	±	0.01
AST (U/L)	195.60	±	26.12	220.20	±	22.37	162.70	±	9.09	213.40	±	21.86	178.20	±	21.36	204.80	±	21.98
ALT (U/L)	57.60	±	9.00	77.70	±	10.96	49.70	±	5.35	73.40	±	9.83	61.10	±	9.88	63.20	±	7.79
ALP (U/L)	43.20	±	2.92	40.60	±	3.03	41.90	±	3.22	48.00	±	7.90	48.10	±	6.03	43.10	±	3.94
Male rats																		
GLU (mg/dl)	209.10	±	8.67	209.40	±	15.59	181.40	±	18.86	219.60	±	14.18	196.40	±	9.49	174.20	±	8.07
BUN (mg/dl)	17.52	±	0.46	18.25	±	0.45	17.29	±	0.63	16.59	±	0.32	16.94	±	0.50	19.62	±	0.31*
CRE (mg/dl)	0.57	±	0.03	0.56	±	0.02	0.54	±	0.02	6.09	±	5.55	0.53	±	0.01	0.61	±	0.02
TP (g/dl)	6.11	±	0.12	5.85	±	0.15	6.19	±	0.09	6.11	±	0.10	6.37	±	0.13	6.25	±	0.18
ALB (g/dl)	3.07	±	0.05	3.03	±	0.06	3.06	±	0.03	3.07	±	0.05	3.10	±	0.04	3.21	±	0.06
T-BIL (mg/dl)	0.20	±	0.03	0.18	±	0.02	0.18	±	0.02	0.14	±	0.02	0.16	±	0.02	0.18	±	0.02
D-BIL (mg/dl)	0.09	±	0.01	0.09	±	0.00	0.10	±	0.01	0.09	±	0.00	0.08	±	0.01	0.18	±	0.07
AST (U/L)	193.00	±	13.81	158.60	±	9.13	152.30	±	13.61*	142.60	±	8.27*	146.80	±	10.34*	201.80	±	22.31
ALT (U/L)	63.20	±	6.35	61.90	±	5.82	54.60	±	5.27	51.30	±	2.86	54.40	±	3.99	83.40	±	9.42*
ALP (U/L)	55.80	±	2.51	53.20	±	2.28	52.90	±	1.59	55.10	±	2.61	61.10	±	2.22	64.90	±	3.40*

A satellite group was given the *B. monnieri* extract at 1,500 mg/kg daily over 270 days followed by no treatment for 28 days

*GLU* glucose, *BUN* blood urea nitrogen, *CRE* creatinine, *TP* total protein, *ALB* albumin, *T-BIL* total bilirubin, *D-BIL* direct bilirubin, *AST* aspartate aminotransferase, *ALT* alanine aminotransferase, *ALP* alkaline phosphatase

Values are expressed as mean ± S.E.M., *n* = 10. *Significantly different from control, *p* < 0.05

**Fig. 3:**
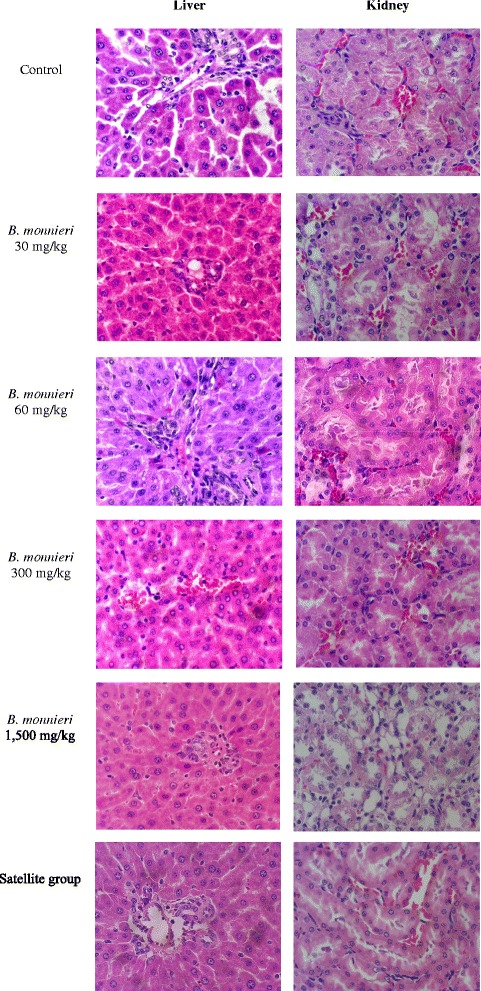
Histopathology of the liver and kidney of rats in chronic toxicity test at 40x magnification (haematoxylin-eosin stain)

No significant histopathological change in the internal organs was found in either the control or the extract-treated rats (Fig. 3).

## Discussion

Toxicity testing is intended to provide information on the safety of an herbal product before further evaluation of its benefits in clinical trials. The appropriate period of administration of the test product to animals is determined by the anticipated period of clinical use in humans [28]. The acute oral toxicity test is used for evaluating any adverse effects appearing within a short time after a single large oral dose of the test substance or after multiple doses given within 24 h. The *B. monnieri* extract at a dose of 5,000 mg/kg was selected for acute toxicity testing as an initial dose of 2,000 mg/kg did not cause any observable signs or symptoms of toxicity. The results showed that *B. monnieri* did not cause death or result in any other signs of toxicity. There was a slight difference in body weight between the control and the treated groups at the beginning of experiment, but it was not statistically significant. However, a small but significant difference in body weight was observed on day 14. The small increase in body weight of the treated animals can still be accepted as normal, as it can occur due to differences in food consumption.

Neither gross nor histopathological abnormalities were observed in any of the internal organs, including the liver and kidney of *B. monnieri*-treated rats. This suggests that *B. monnieri* extract is nontoxic at a dose of 5,000 mg/kg. The dose of *B. monnieri* extract used in this study is approximately 1,000 times higher than that generally used in humans (5 mg/kg/day). This indicates that *B. monnieri* is quite safe for human use.

A chronic toxicity study normally takes 9 to 12 months as some substances may not display toxicity immediately, but it may appear after repeated exposure. According to WHO guidelines, the period of testing administration in animals should be based on the expected period of clinical use of that substance in humans. The repeated oral administration of the test substance for 9-12 months in animals has been suggested to be comparable to administration for more than 6 months in humans [28].

The objective of chronic toxicity studies is to characterize the profile of the impact of prolonged and repeated exposure to a substance in a mammalian species over a significant portion of the average life span of that species. The study evaluates the impact on target organs including possible accumulation of the substance. The results obtained can then be used as supporting data for designing further clinical trials. The dosage of plant extract for chronic toxicity testing in rats in this study was calculated according to Reagan-Shaw et al. [30]. The *B. monnieri* extract at doses of 30, 60, 300 and 1,500 mg/kg used in this study are equivalent to 6, 12, 60, and 300 times of the usual *B. monnieri* dose in humans, respectively.

In this study, no changes in animal behavior or any toxic signs were observed in the *B. monnieri*-treated rats. Body weight and internal organ weight were the main parameters used in the assessment of toxicity. Reduction of those two parameters can be used as a sensitivity index of toxicity [31–33]. In this study, the animals were weighed daily throughout the experimental period. Although the weight of some groups of rats decreased or increased slightly, the differences were very small and remained within normal limits. Monitoring the health of the animals during the entire 270 day period found no signs of morbidity or diseases. Rats of both sexes appeared healthy as evidenced by the normal appearance of their general behavior, respiratory patterns, cardiovascular signs, motor activities, reflexes and lack of abnormal changes of skin or fur.

The internal organs were weighed and examined for gross pathology. Some test substances may harm tissues at the cellular level but not show any observable abnormalities. For this reason histopathological examination should be carried out to identify any cellular damage of the internal organs or tissues. In this study, internal organs including the brain, lungs, heart, liver, kidney, pancreas, spleen, stomach, duodenum, small intestine, and sex organs were examined. The examination found no significant histopathological changes in these organs or tissues. Although some statistically significant but minor differences in the body weight and the weight of some of the internal organs were observed, the values of those parameters were in normal range.

Hematological examination is one of the methods used to evaluate aspects of health status that may not be visible during physical examination [34]. This is important because the hematopoietic system is very sensitive to toxic substances. It can be affected by the ingestion of some toxic plants [35]. In particular, hematological parameters can provide important information about bone marrow activity as well as about intravascular effects such as hemolysis and anemia [36]. Morphological examination of RBC and WBC can identify their production or destruction. BASA, EOS, LYMP, MONO, and PMN are also used to assess the effect of test substances on immune systems [36–38]. Our results found significant differences in some hematological parameters, but values remained within normal limits [39].

Clinical blood chemistry examination was performed to quantify any toxic effects on pancreas function (GLU), kidney function (BUN, CRE) and liver function (SGOT, SGPT, ALP, TP, ALB, T-BIL and D-BIL). Plasma GLU is monitored to demonstrate the effect of a test substance on glucose metabolism. In terms of kidney function, the most common cause of acute or chronic renal failure is elevated BUN and increased CRE. Liver function is measured using a blood test which evaluates various functions of the liver, e.g., metabolism, detoxification, storage, and production of several vital protein components of blood plasma. Conditions commonly associated with testing for abnormalities of liver function include gallbladder disease, hepatitis, fatty liver, cirrhosis, infectious mononucleosis and alcoholism [38, 40]. In this study, significant differences in some clinical blood chemistry values were observed in both sexes between the experimental and the control groups. However, the alteration of these parameters was minor and remained within the normal range [41]. The data suggest that *B. monnieri* extract does not alter the function of the pancreas, liver or kidney.

The satellite-treated group was assessed for reversibility of the toxic effects of the test substance and for the occurrence of delayed toxic effects. In this study, the satellite-treated group received *B. monnieri* extract at a dose of 1,500 mg/kg/d for 270 days. The rats were then kept for an additional 28 days post treatment. No toxic signs or symptoms or any abnormalities were detected.

Taken together, all of our results from both the acute and the chronic toxicity tests indicate that the *B. monnieri* extract is fairly non toxic for chronic use.

## Conclusion

*B. monnieri* extract did not cause any signs of toxicity or any other symptoms in the acute and chronic oral toxicity tests of both female and male rats. That indicates that *B. monnieri* extract is relatively non-toxic. Further study regarding the toxicology of this extract should be conducted in non-rodent species or in humans.

## Abbreviations

ALB, albumin; ALP, alkaline phosphatase; ALT, alanine aminotransferase; AST, aspartate aminotransferase; BASO, basophil; BUN, blood urea nitrogen; CRE, creatinine; D-BIL, direct bilirubin; EOS, eosinophil; GLU, glucose; HB, hemoglobin; HCT, hematocrit; LYMP, lymphocyte; MCH, mean corpuscular hemoglobin; MCHC, mean corpuscular hemoglobin concentration; MCV, mean corpuscular volume; MONO, monocyte; PLT, platelet; PMN, neutrophil; RBC, Red blood cell count; T-BIL, total bilirubin; TP, total protein; WBC, white blood cell count
